# CCAAT/enhancer-binding protein δ (C/EBPδ) aggravates inflammation and bacterial dissemination during pneumococcal meningitis

**DOI:** 10.1186/s12974-015-0309-5

**Published:** 2015-05-10

**Authors:** Mercedes Valls Serón, JanWillem Duitman, Madelijn Geldhoff, JooYeon Engelen-Lee, Stefan R Havik, Matthijs C Brouwer, Diederik van de Beek, C Arnold Spek

**Affiliations:** Department of Neurology, Center of Infection and Immunity Amsterdam (CINIMA), Academic Medical Center, University of Amsterdam, Meibergdreef 9, PO Box 22660, 1100DD Amsterdam, The Netherlands; Center for Experimental and Molecular Medicine (CEMM), Academic Medical Center, Meibergdreef 9, 1105 AZ Amsterdam, The Netherlands

**Keywords:** Experimental meningitis, C/EBPδ, Infection, *Streptococcus pneumoniae*

## Abstract

**Background:**

The prognosis of bacterial meningitis largely depends on the severity of the inflammatory response. The transcription factor CAAT/enhancer-binding protein δ (C/EBPδ) plays a key role in the regulation of the inflammatory response during bacterial infections. Consequently, we assessed the role of C/EBPδ during experimental meningitis.

**Methods:**

Wild-type and C/EBPδ-deficient mice (C/EBPδ^−/−^) were intracisternally infected with *Streptococcus pneumoniae* and sacrificed after 6 or 30 h, or followed in a survival study.

**Results:**

In comparison to wild-type mice, C/EBPδ^−/−^ mice showed decreased bacterial loads at the primary site of infection and decreased bacterial dissemination to lung and spleen 30 h after inoculation. Expression levels of the inflammatory mediators IL-10 and KC were lower in C/EBPδ^−/−^ brain homogenates, whereas IL-6, TNF-α, IL-1β, and MIP-2 levels were not significantly different between the two genotypes. Moreover, C/EBPδ^−/−^ mice demonstrated an attenuated systemic response as reflected by lower IL-10, IL-6, KC, and MIP-2 plasma levels. No differences in clinical symptoms or in survival were observed between wild-type and C/EBPδ^−/−^ mice.

**Conclusion:**

C/EBPδ in the brain drives the inflammatory response and contributes to bacterial dissemination during pneumococcal meningitis. C/EBPδ does, however, not affect clinical parameters of the disease and does not confer a survival benefit.

## Introduction

Bacterial meningitis remains an important cause of mortality and morbidity worldwide, despite the implementation of vaccination strategies, effective antibiotic therapy and adjunctive dexamethasone treatment [1,2]. Pneumococcal meningitis is the most common and most severe form of meningitis: 16% to 35% of the patients die and up to 50% of survivors suffer from long-term sequelae, including hearing loss, cognitive impairment, and focal neurological deficits [3,4]. The pathophysiology of bacterial meningitis is characterized by a strong inflammatory response in the subarachnoid space, and this host inflammatory response seems to cause adverse events during bacterial meningitis [5,6]. The severity of the inflammatory response largely has been shown to determine the prognosis in experimental pneumococcal meningitis [7] and novel therapeutic agents may thus target the inflammatory response. However, the inflammatory response during meningitis is complex and has only been partially elucidated.

CAAT/enhancer-binding protein δ (C/EBPδ), a member of the C/EBP family of transcription factors that is upregulated during the acute phase response, recently emerged as an essential player in the inflammatory response to bacterial infections [8]. C/EBPδ levels rapidly increase after pro-inflammatory stimuli such as lipopolysaccharide (LPS), interleukin (IL)-1, IL-6, interferon γ (IFN-γ), and tumor necrosis factor-α (TNF-α) [9-11]. Reciprocally, C/EBPδ enhances cytokine production and C/EBPδ-induced inflammation contributes to elimination of bacteria during infectious disease. C/EBPδ was shown to limit bacterial dissemination and prolong survival during a lethal model of *Escherichia coli*-induced peritonitis and during *Klebsiella pneumoniae*-induced pulmonary infection [12,13]. During *Streptococcus pneumoniae*-induced pulmonary infection, however, C/EBPδ exaggerated bacterial dissemination and wild-type mice succumbed earlier to the disease as compared to C/EBPδ^−/−^ mice [14]. Furthermore, C/EBPδ did not affect disease progression during non-lethal models of *E. coli*-induced peritonitis or urinary tract infection [12,15]. C/EBPδ therefore seems to play a complex and potential dual role during infectious disease most likely depending on the causing pathogen, the severity of the infection and the infection site. In the present study, we assessed the role of C/EBPδ in experimental pneumococcal meningitis.

## Materials and methods

### Mice

Eight- to 12-week-old female C57BL/6 mice were obtained from Charles River (Sulzfeld, Germany), whereas C/EBPδ^−/−^ mice, generated as described previously [16], were bred and maintained at the animal facility at the Academic Medical Center of Amsterdam. The mice were kept to a controlled 12 h light/dark cycle and food and water were provided *ad libitum*. All experiments were approved by the Institutional Animal Care and Use Committee of the Academic Medical Center, Amsterdam.

### Experimental pneumococcal meningitis model

Pneumococcal meningitis was induced by intracisternal inoculation with *S. pneumoniae* (serotype 3, American Type Culture Collection #6303; Rockville, MD) as described previously [17]. In brief, wild-type and C/EBPδ^−/−^ mice (*n* = 12 per group) were inoculated with 1 μL bacterial suspension containing 1 × 10^4^ CFU *S. pneumoniae* into the cisterna magna under isoflurane anesthesia. Six mice per group inoculated with sterile saline were used as controls. Immediately after intracisternal inoculation, mice were assessed for neurologic damage as a result of the puncture, and if present, these mice were excluded from further analysis. Clinical signs of meningitis were scored at 24 and 30 h post infection as previously described [17]. At 6 and 30 h post infection, mice were anesthetized by intraperitoneal injection of 190 mg/kg ketamine (Eurovet Animal Health, Bladel, The Netherlands) and 0.3 mg/kg dexmedetomidine (Pfizer Animal Health, Capelle aan den Ijssel, The Netherlands) followed by cardiac puncture for blood collection and perfusion of organs with sterile isotonic saline *via* the left ventricle. CSF was collected by puncture of the cisterna magna. Brain, lung, and spleen were harvested, placed on ice, processed, and stored as described before [17]. EDTA blood was centrifuged at 2,000×*g* for 15 min. Plasma was stored at −80°C for further analysis.

### Determination of cytokines and chemokines

The cytokines IL-1β, IL-6, IL-10, and TNF-α and the chemokines KC and MIP-2 were determined in plasma and brain homogenates by Luminex technology using a mouse Bioplex kit (Bio-Rad Laboratories, Veenendaal, The Netherlands) as described before [17].

### Organ damage markers

Plasma aspartate aminotransferase, alanine aminotransferase, and lactate dehydrogenase levels were determined as described before [18].

### Murine histopathology and immunohistochemistry

Histopathology was performed on the right cerebral hemisphere fixed in 4% paraformaldehyde and paraffin embedded. Coronal 5-μm-thick sections of the right hemisphere were cut for subsequent staining with hematoxylin and eosin (HE) according to standard procedures. Tissue sections were stained for C/EBPδ using immunohistochemical procedures, as described previously [14].

### Real-time PCR

Total RNA was extracted from snap frozen murine brain homogenates using TriPure reagent (Sigma-Aldrich, St-Louis, MO, USA). For complementary DNA (cDNA) synthesis, RNA was treated with RQ1 RNase-free DNase (Promega, Leiden, The Netherlands) and reverse transcribed with SuperScript II Reverse Transcriptase and randomhexamers (Life Technologies, Bleiswijk, The Netherlands). The real-time polymerase chain reaction (RT-PCR) measurement of individual cDNAs was performed on a Bio-Rad MyiQ Single-Color RT-PCR Detection System using the Bio-Rad iQ SYBR Green Supermix (Bio-Rad Laboratories, Hercules, CA, USA). The *c*/*ebpδ* and Non-POU-domain containing octamer binding protein (NoNo, housekeeping gene), primers were described previously [14,19]. The expression data were normalized to the NoNo reference gene.

### Statistical analysis

Differences between groups were analyzed using the Mann-Whitney *U*-test. For the survival experiment, a Kaplan-Meier analysis was performed using the log-rank test. For all analyses, a *P* value of <0.05 was considered to be a significant difference.

## Results

### C/EBPδ expression is increased during pneumococcal meningitis

**Figure 1:**
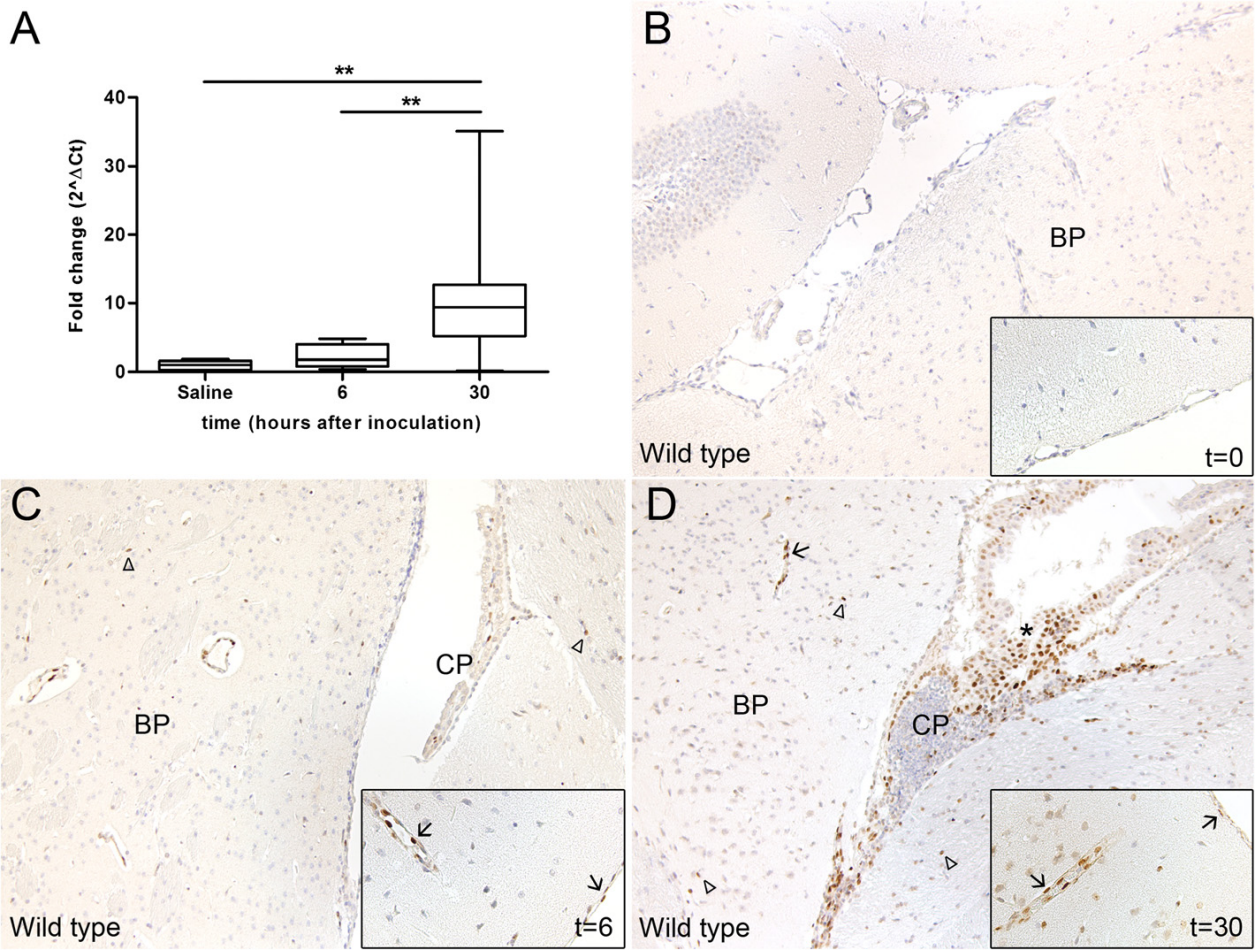
Pneumococcal meningitis induces C/EBPδ expression. *c*/*ebpδ* mRNA **(A)** and protein expression in wild-type brain tissue **(B,** **C,** **D)** at different time points after intracisternal inoculation with *S. pneumoniae*. Arrows indicate epithelial cells expressing C/EBPδ in vessels of the brain parenchyma (BP) and meningeal cells. Arrowheads indicate glial cells in brain parenchyma expressing C/EBPδ (BP). Asterisk indicate C/EBPδ positive cells in the choroid plexus (CP). Original magnification ×10, inset ×20. Data are expressed as median with interquartile ranges and 5% to 95% percentiles (*n* = 6 for controls; *n* = 12 at both *t* = 6 and *t* = 30 h post infection). **P < 0.01.

To obtain insight into the expression of C/EBPδ during meningitis caused by *S. pneumoniae*, we measured *c*/*ebpδ* mRNA levels in brain tissue from wild-type mice intracisternally inoculated with 1 × 10^4^ CFU. *c*/*ebpδ* mRNA expression was low in uninfected brain, slightly increased at 6 h after *S. pneumoniae* inoculation, although this did not reach statistical difference, whereas c/ebpδ levels were about tenfold increased at 30 h after infection (Figure 1A). Immunohistochemistry confirmed the mRNA data, and showed increased C/EBPδ expression in the brains of infected mice over time (Figure 1B,C,D). C/EBPδ was strongly expressed in the endothelium (both in brain parenchyma and meninges), ependymal and choroid plexus, and, to a lesser extent, in glia and arachnoidal cells.

### C/EBPδ deficiency limits bacterial dissemination during pneumococcal meningitis

**Figure 2:**
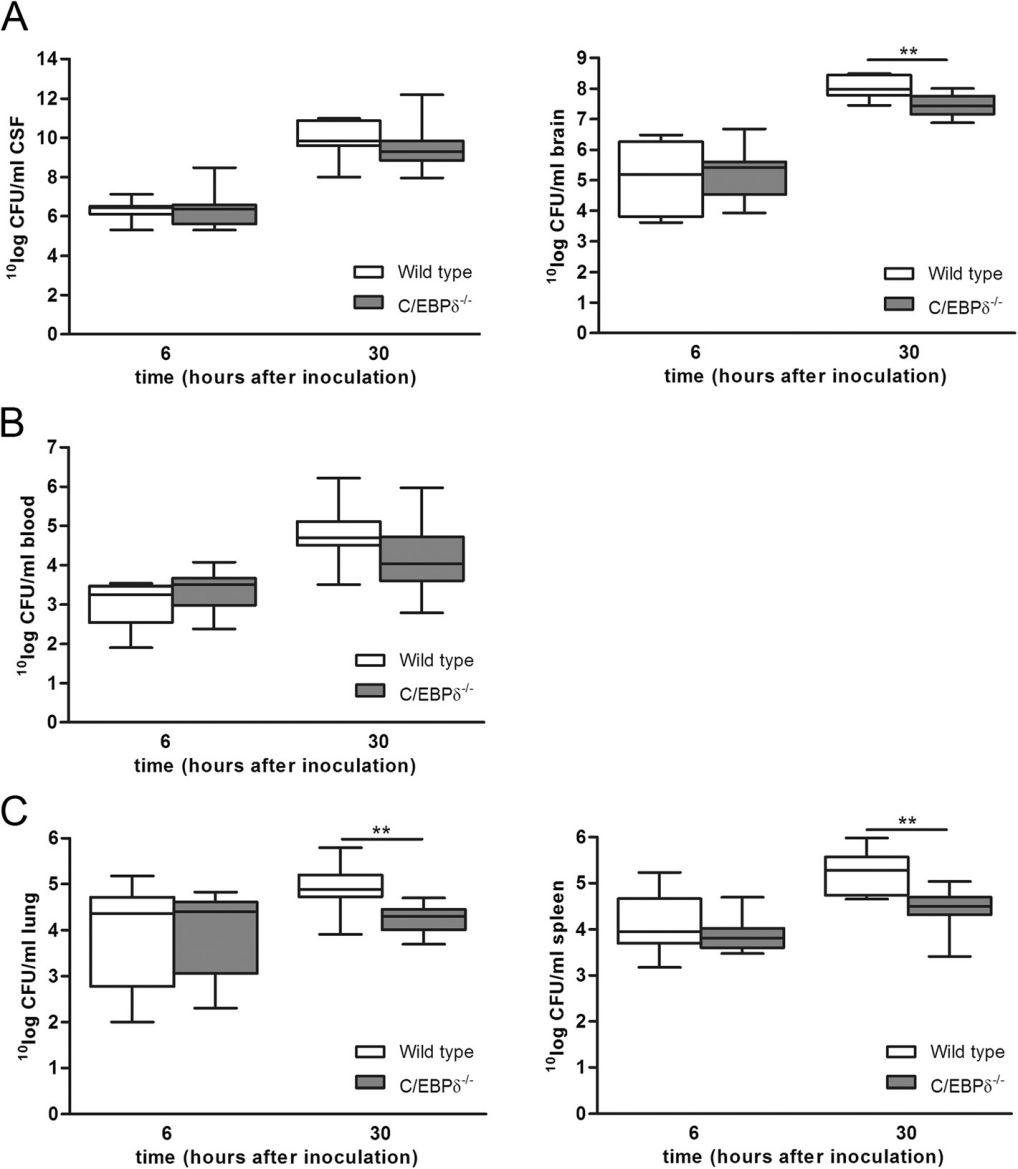
C/EBPδ^−/−^ mice have less bacterial outgrowth in brain, lung, and spleen at 30 h after pneumococcal meningitis. Bacterial outgrowth in CSF and brain homogenates **(A)**, whole blood **(B)**, lung homogenates and spleen homogenates **(C)** of wild-type (open boxes) and C/EBPδ^−/−^ (gray boxes) 6 and 30 h after infection. Data are expressed as median with interquartile ranges and 5% to 95% percentiles (*n* = 12 per group). **P < 0.01 compared to wild-type mice.

To determine the role of C/EBPδ on bacterial dissemination during pneumococcal meningitis, we determined bacterial loads at 6 and 30 h after infection. At 6 h after inoculation, bacterial outgrowth in both CSF and brain homogenates from C/EBPδ^−/−^ mice was similar to that observed in wild-type mice (Figure 2A). Dissemination of bacteria into the bloodstream and to distal organs was also similar in both groups (Figure 2B,C). At 30 h after inoculation, bacterial loads were significantly increased compared to the 6-h time point. C/EBPδ^−/−^ mice had lower bacterial counts in brain (median 9.5 × 10^7^ CFU/ml vs. 2.7 × 10^7^ CFU/ml for wild-type and C/EBPδ^−/−^ mice, respectively, *P* = 0.02) consistent with a trend toward lower bacterial loads in blood of C/EBPδ^−/−^ mice (median 5.0 × 10^4^*vs*. 1.1 × 10^4^ for wild-type and C/EBPδ^−/−^ mice, respectively, *P* = 0.06) and lower bacterial numbers in lung (median 7.7 × 10^4^*vs*. 2.0 × 10^4^ for wild-type and C/EBPδ^−/−^ mice, respectively, *P* = 0.001) and spleen (median 1.9 × 10^5^*vs*. 3.1 x 10^4^ for wild-type and C/EBPδ^−/−^ mice, respectively, *P* = 0.0004) of C/EBPδ^−/−^ mice. In CSF, bacterial counts were similar between wild-type and C/EBPδ^−/−^ mice (median 7.0 × 10^9^*vs*. 2.0 × 10^9^ for wild-type and C/EBPδ^−/−^ mice, respectively, *P* = 0.20). Overall, these results show that C/EBPδ deficiency limits bacterial growth and dissemination during pneumococcal meningitis at the later stages of the disease.

### C/EBPδ deficiency attenuates cytokine and chemokine production during pneumococcal meningitis

**Figure 3:**
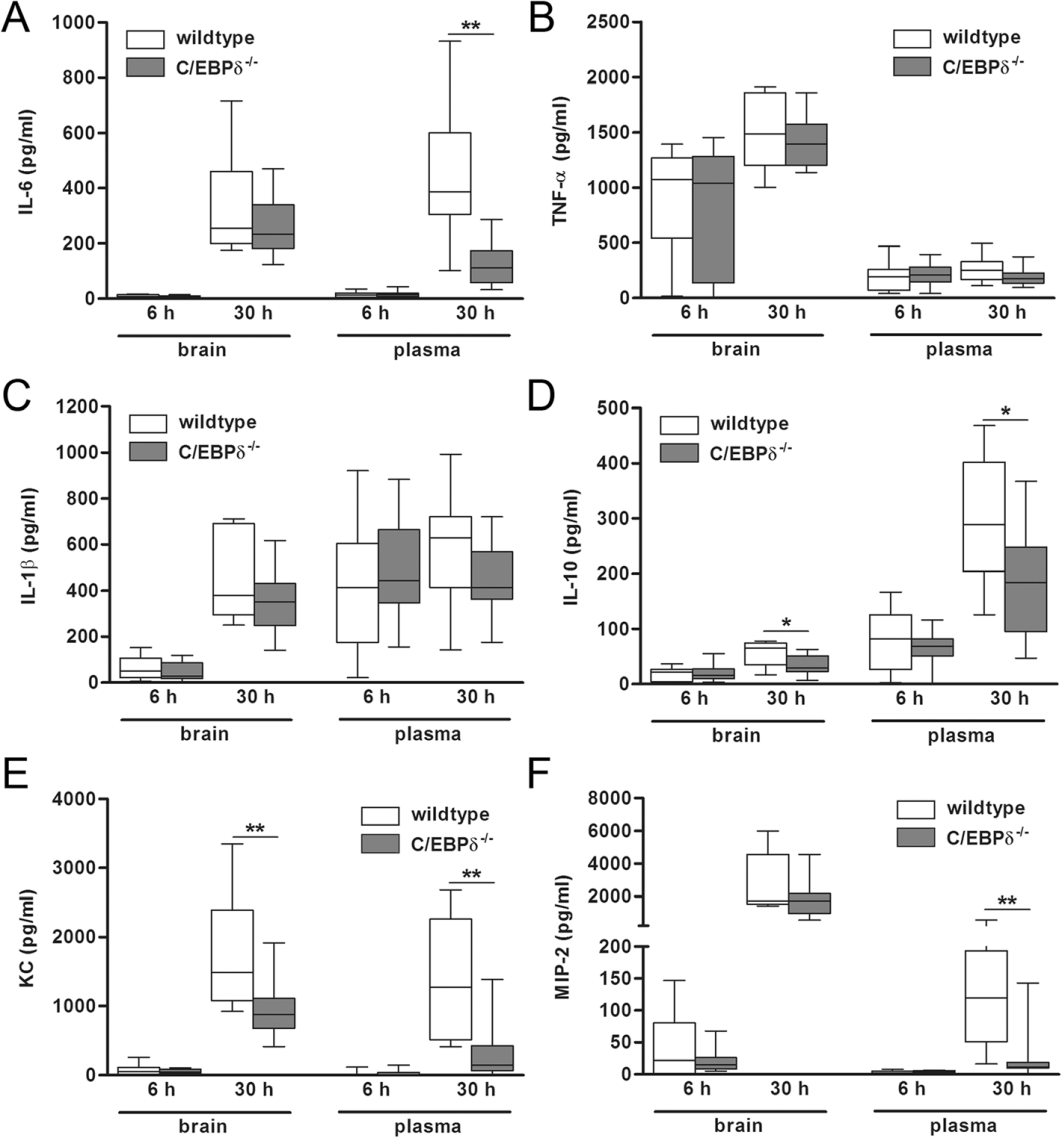
C/EBPδ deficiency modifies the inflammatory response during pneumococcal meningitis. Cytokine **(A,** **B,** **C,** **D)** and chemokine **(E,** **F)** levels in brain homogenates and plasma of wild-type (open boxes) and C/EBPδ^−/−^ (gray boxes) mice 6 and 30 h after infection. Data are expressed as median with interquartile ranges and 5% to 95% percentiles (*n* = 11 to 12 per group). *P < 0.05 compared to wild-type control mice.

To determine whether C/EBPδ influences inflammation associated with pneumococcal meningitis, we assessed the impact of C/EBPδ deficiency on the inflammatory host response. At 6 h post infection, the extent of inflammation both locally in the brain and systemically in plasma did not differ between both genotypes (Figure 3). At 30 h post infection, IL-10 and KC levels were lower in C/EBPδ^−/−^ brain homogenates, whereas brain IL-6, TNF-α, IL-1β, and MIP-2 levels were not significantly different between the two genotypes. C/EBPδ deficiency attenuated the systemic inflammatory response as reflected by lower IL-10, IL-6, KC, and MIP-2 levels in plasma of C/EBPδ^−/−^ mice as compared to wild-type mice.

To assess whether the inflammatory response is a mere reflection of the bacterial load, cytokine/chemokine levels were correlated with the bacterial burden in brains of wild-type and C/EBPδ^−/−^ mice. As shown in Table 1, cytokine/chemokine levels and bacterial counts were strongly correlated in wild-type mice with correlation coefficients ranging from 0.39 to 0.84. Only TNF-α, IL-1β, and MIP-2 levels correlated with bacterial loads in C/EBPδ^−/−^ mice (correlation coefficients of 0.44 to 0.68), whereas levels of IL-6, IL-10, and KC did not show any correlation.

**Table 1:** **Correlation of cytokine**/**chemokine levels with bacterial outgrowth of wild**-**type and C**/**EBPδ** ^−/−^ **mice upon meningitis**

	**Brain** **(30 h after inoculation)**			
	**Wild type**		**C/** **EBPδ** ^**−/−**^	
	***(n*** **=** **11)**		***(n*** **=** **12)**	
**Cytokines**	**(** ***r*** ^**2**^ **)**	***P*** **value**	**(** ***r*** ^**2**^ **)**	***P*** **value**
**IL**-**6**	0.7223	*0.0009*	0.0499	0.4853
**TNF**-**α**	0.3922	*0.0393*	0.4356	*0.0271*
**IL**-**1β**	0.3922	*0.0393*	0.4356	*0.0271*
**IL**-**10**	0.5679	*0.0074*	0.0405	0.5303
**Chemokines**				
**KC**	0.8320	<*0.0001*	0.2704	0.1011
**MIP**-**2**	0.8405	<*0.0001*	0.6779	*0.0010*

Data were obtained using a Pearson correlation analysis and presented as linear regression values for each group (*r* ^2^) and concomitant P values. Significant correlations (*P* < 0.05) are in italic. Non-significant values are shown in gray.

### C/EBPδ does not affect clinical outcome of pneumococcal meningitis

**Figure 4:**
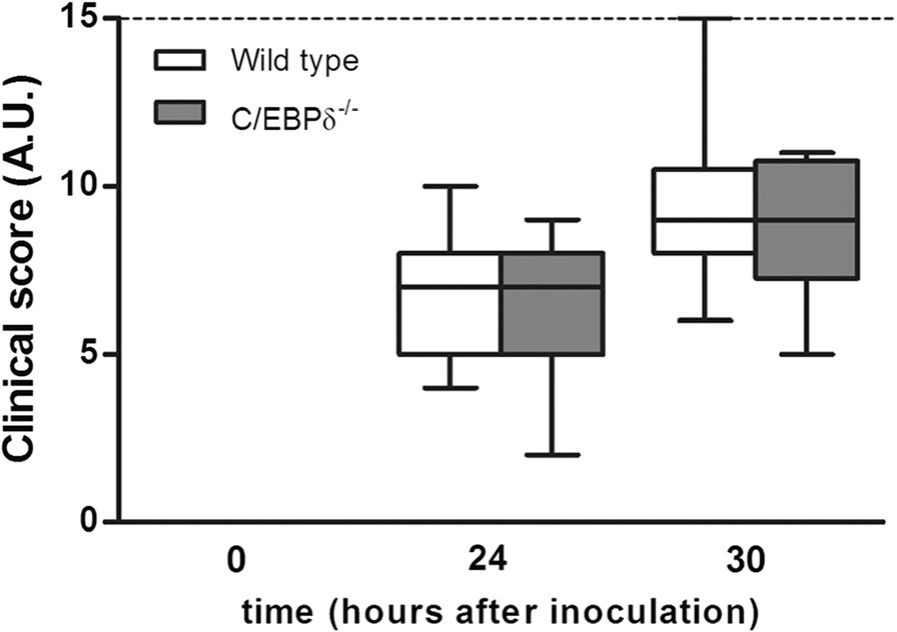
Effect of C/EBPδ deficiency on the clinical status during pneumococcal meningitis. Clinical scores of wild-type and C/EBPδ^−/−^ mice at different time points during meningitis. A.U. = arbitrary units; dashed line indicates the maximal score of 15.

Severe bacterial meningitis leads to several clinical (neurological) symptoms such as disorientation, paralysis, and an increased breathing frequency. In order to assess whether the diminished bacterial loads and the reduced cytokine levels in C/EBPδ^−/−^ mice would limit disease progression, these specific clinical symptoms were scored at 24 and 30 h post infection. As shown in Figure 4, clinical scores significantly increased over time during infection reaching the maximum score of 15 in some mice at 30 h post infection. This increase was, however, similar in wild-type and C/EBPδ^−/−^ mice.

**Figure 5:**
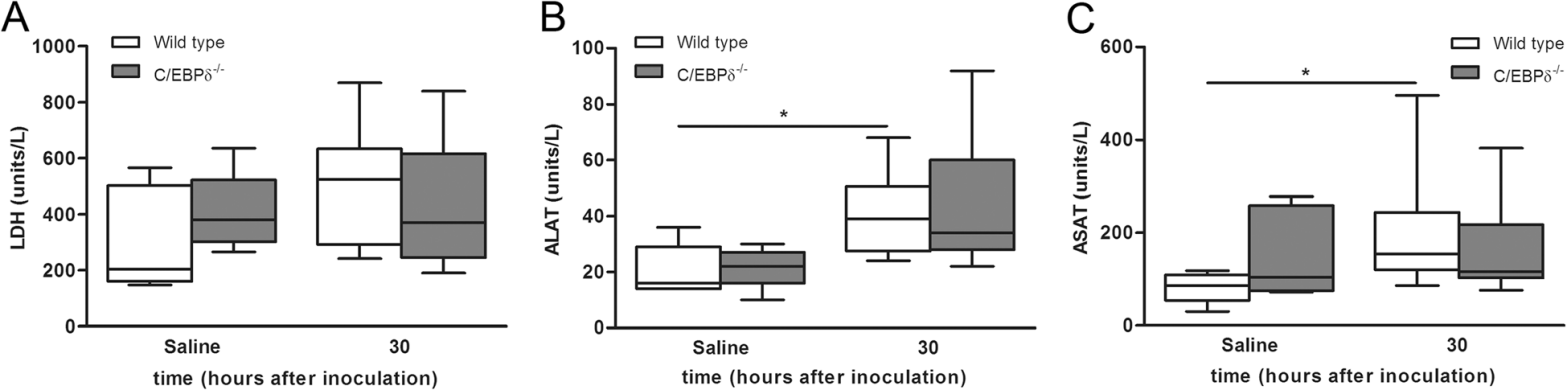
Effect of C/EBPδ deficiency on organ damage during pneumococcal meningitis. Levels of general tissue injury marker lactate dehydrogenase (LDH; **A**), liver injury markers alanine transaminase (ALAT; **B**) and aspartate transaminase (ASAT; **C**) in plasma of wild-type (open boxes) and C/EBPδ^−/−^ (gray boxes) 30 h after infection. Data are expressed as median with interquartile ranges and 5% to 95% percentiles. *P < 0.05 compared to wild-type control mice.

To assess the effect of C/EBPδ on meningitis-induced general markers of tissue injury, we next assessed lactate dehydrogenase (LDH), aspartate transaminase (ASAT), and alanine transaminase (ALAT) levels. As shown in Figure 5A,B,C, ASAT and ALAT levels significantly increased in wild-type animals during *S. pneumoniae* infection, whereas LDH was not induced during meningitis. ASAT and ALAT levels increased to similar extent in C/EBPδ^−/−^ mice.

**Figure 6:**
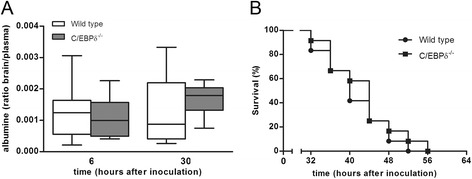
Effect of C/EBPδ deficiency on blood-brain barrier status and survival. Brain/plasma albumin ratio in wild-type (open boxes) and C/EBPδ^−/−^ (gray boxes) 30 h after infection. Data are expressed as median with interquartile ranges and 5% to 95% percentiles. *P < 0.05 compared to wild-type control mice **(A)**. Survival of wild-type (black circle) and C/EBPδ^−/−^ mice (black square) after induction of pneumococcal meningitis (*n* = 12 mice per group; **B**).

During bacterial meningitis, destruction of the blood-brain barrier is related to disease progression and subsequent survival [7]. In order to get insight into the blood-brain barrier status during meningitis, the brain/plasma albumin ratio was determined for wild-type and C/EBPδ^−/−^ mice. As shown in Figure 6A, the albumin ratio was not different between the two genotypes suggesting that C/EBPδ deficiency does not affect the disruption of the blood-brain barrier during meningitis.

We next assessed whether the observed C/EBPδ-dependent increased bacterial loads and cytokine levels affected survival. In the survival study, the first mice succumbed at 32 h post infection and all mice died within 56 h post infection (Figure 6B). There was no difference in survival between wild-type and knockout mice.

## Discussion

This study shows that C/EBPδ expression in the brain is induced during pneumococcal meningitis and that C/EBPδ contributes to bacterial colonization of the brain and dissemination to distant organs during disease progression. Although C/EBPδ potentiates the pro-inflammatory response during meningitis, it does not modify clinical parameters of disease severity nor does it affect mortality.

The observed increase in C/EBPδ expression during meningitis is in line with increased C/EBPδ expression in alternative infectious disease models. C/EBPδ expression was previously shown to be increased during peritonitis [12], pneumonia [14,13], and urinary tract infection [15]. However, the consequence of increased C/EBPδ expression in infectious disease seems not to be straightforward as it correlates with increased survival in some models and with decreased survival in others. During *E. coli*-induced peritonitis, C/EBPδ limits bacterial growth and C/EBPδ^−/−^ mice succumb due to the infection at an earlier time point as compared to wild-type mice [12]. Similar to these findings, increased C/EBPδ expression during *K. pneumoniae*-induced pulmonary infection was found to contribute to improved survival as evident from reduced survival of C/EBPδ^−/−^ mice [13]. In contrast, increased C/EBPδ expression during urinary tract infection did not affect disease progression nor outcome [15]. C/EBPδ expression also contributed to bacterial outgrowth and dissemination leading to prolonged survival of C/EBPδ^−/−^ mice during *S. pneumoniae*-induced pulmonary infection [14]. This is in line with our results showing that bacterial dissemination was hampered in C/EBPδ^−/−^ mice as compared to wild-type mice during pneumococcal meningitis leading to a lower bacterial burden in blood and peripheral organs. C/EBPδ-driven bacterial dissemination during meningitis did, however, not modify clinical parameters of meningitis and did not affect overall survival, whereas C/EBPδ-driven dissemination during pneumonia was detrimental. These data underscore the fact that C/EBPδ-driven dissemination plays a divergent role during infectious disease leading to different outcome of the disease.

In our study, we confirm the established role of C/EBPδ as activator of the inflammatory response, reflected by the reduction in IL-10 and KC levels in the brain and IL-6, IL-10, KC, and MIP-2 in plasma during pneumococcal meningitis. The reduced levels of inflammatory mediators in C/EBPδ^−/−^ mice during meningitis may be a direct consequence of C/EBPδ-driven transcriptional activity but may also be a mere reflection of the bacterial burden. Indeed, the extent of inflammation closely follows the bacterial burden during experimental pneumococcal pneumonia [20,21], although such a correlation has not been established for pneumococcal meningitis yet. Here, we show for the first time that during meningitis cytokine and chemokine levels also positively correlate with the bacterial burden in the brain of wild-type mice (id est IL-6, TNF-α, IL-1β, IL-10, KC, and MIP-2). However, such a correlation is not observed for IL-6, IL-10, and KC in C/EBPδ^−/−^ mice, which suggests that C/EBPδ directly drives transcriptional activity of these inflammatory mediators during meningitis. This is in line with previous reports showing that C/EBPδ is involved in the regulation of IL-6 [12,22] and IL-10 [23] after LPS stimulation, although C/EBPδ suppresses IL-10 expression in dendritic cells of the central nervous system in an animal model of multiple sclerosis [24]. Moreover, TNF-α and MIP-2 levels are not dependent on C/EBPδ during pneumococcal meningitis, although these cytokines have been shown to be regulated by C/EBPδ during LPS-induced models of acute lung injury [22] and endotoxemia [25]. We currently have no proper explanation for these seeming contradictive results, although the role of C/EBPδ in the inflammatory response might strongly depend on the underlying disease.

Our results show that the C/EPBδ-mediated inflammatory response is not a major mechanism explaining poor outcome in pneumococcal meningitis. We did not find differences in survival nor clinical parameters between wild-type and C/EBPδ^−/−^ mice. Apparently, the lower bacterial burden in combination with the lower inflammatory response in C/EBPδ^−/−^ did not affect clinical parameters of meningitis. In a previous study, reduced bacterial loads did correlate with improved survival during *S. pneumoniae*-induced meningitis. Formyl peptide receptor (FPR)-1 or -2 deficient mice had higher bacterial titers than wild-type mice after pneumococcal meningitis, and wild-type mice lived significantly longer than both knockout strains [26]. Although we do not have a clear explanation for these different results at the moment, gender differences (male mice in the FPR study and female mice in our study) and/or used pathogens (type 2 *vs*. type 3 *S. pneumoniae* in the FPF and our study, respectively) may explain at least part of the observed differences. A possible explanation for the discrepancy between clinical outcome and decreased bacterial burden and lower inflammatory response observed in this study could be that C/EBPδ also plays a crucial role in neuroprotective processes. Indeed, levels of IL-10, an important anti-inflammatory mediator with a protective function in pneumococcal meningitis [7], are significantly decreased in C/EBPδ^−/−^ mice as compared to wild-type mice. Although reduced IL-10 levels may thus explain the lack of effect on clinical parameters, alternative, currently unknown, neuroprotective properties of C/EBPδ in the brain cannot be excluded.

Our study has several limitations. First, we only studied a single dose of a single serotype pneumococcus. As the virulence of different pneumococcal serotypes varies widely, experimental meningitis due to other serotypes may yield different results. Furthermore, using different inoculum sizes may show more subtle differences. However, our model uses the most common serotype found in patients with pneumococcal meningitis, and the used inoculation size results in a mortality similar to that observed in patients [17,4]. Therefore, we feel that our model provides sufficient information to conclude there is a lack of effect on survival of C/EBPδ. Second, we did not treat our mice with antibiotic therapy, and therefore our model does not reflect the clinical situation. However, the aim of our study was to further study the role of C/EBPδ in the pathophysiology of pneumococcal meningitis, which is best evaluated in untreated models.

In conclusion, we show that C/EBPδ expression increases in the brain of mice during meningitis and that C/EBPδ^−/−^ expression contributes to bacterial growth and/or dissemination and induces the inflammatory response. C/EBPδ expression did not affect clinical parameters nor did it change survival suggesting that C/EBPδ plays complex role in *S. pneumoniae*-induced meningitis.
